# Implementing a successful tuberculosis programme within primary care services in a conflict area using the stop TB strategy: Afghanistan case study

**DOI:** 10.1186/1752-1505-8-3

**Published:** 2014-02-07

**Authors:** Khaled Seddiq, Donald A Enarson, Karam Shah, Zaeem Haq, Wasiq M Khan

**Affiliations:** 1 National TB Control Programme, Darul-aman, Kabul, Afghanistan; 2 International Union against Tuberculosis and Lung Disease, Paris, France; 3 Stop-TB, Kabul, Afghanistan; 4 Health Services Academy, Islamabad, Pakistan; 5 WHO Regional Office for the Eastern Mediterranean, Cairo, Egypt

**Keywords:** Afghanistan, Conflict, Tuberculosis, Implementation, Sustainability

## Abstract

**Introduction:**

Afghanistan has faced health consequences of war including those due to displacement of populations, breakdown of health and social services, and increased risks of disease transmission for over three decades. Yet it was able to restructure its National Tuberculosis Control Programme (NTP), integrate tuberculosis treatment into primary health care and achieve most of its targets by the year 2011. What were the processes that enabled the programme to achieve its targets? More importantly, what were the underpinning factors that made this success possible? We addressed these important questions through a case study.

**Case description:**

We adopted a processes and outcomes framework for this study, which began with examining the change in key programme indicators, followed by backwards tracing of the processes and underlying factors, responsible for this change. Methods included review of the published and grey literature along with in-depth interviews of 15 key informants involved with the care of tuberculosis patients in Afghanistan.

**Discussion and evaluation:**

TB incidence and mortality per 100,000 decreased from 325 and 92 to 189 and 39 respectively, while case notification and treatment success improved during the decade under study. Efficient programme structures were enabled through high political commitment from the Government, strong leadership from the programme, effective partnership and coordination among stakeholders, and adequate technical and financial support from the development partners.

**Conclusions:**

The NTP Afghanistan is an example that public health programmes can be effectively implemented in fragile states. High political commitment and strong local leadership are essential factors for such programmes. To ensure long-term effectiveness of the NTP, the international support should be withdrawn in a phased manner, coupled with a sequential increase in resources allocated to the NTP by the Government of Afghanistan.

## Background

Armed conflicts bring death and disability both on and off the battlefield. The health consequences off the combat zone include those due to displacement of populations, breakdown of health and social services, and increased risks of disease transmission [1-3]. According to the United Nations Development Programme (UNDP), 35 countries out of which 60% belonged to the resource-poor category, faced conflict or entered into post-conflict phase during 1989–2008 [4]. Resource-poor countries caught in conflict situations face monumental social and health challenges including high morbidity and mortality, economic downtrends, human and capital flight and destruction of infrastructure [4]. Between a quarter and a half of the countries in post-conflict situation also experience a renewal of the conflict, more likely among low-income countries [5].

Afghanistan is a low-income country that has experienced all the three phases: there have been conflicts, post-conflicts, as well as renewal of conflicts. For decades the country has been in the situation of war, crippled law and order, uncertainty, and frequent migration of populations [6]. The health system was already badly affected by poor policy and planning before the 2001 war. The war resulted in severe damage to the health infrastructure including hospitals and clinics across the country [7]. Health services to combat tuberculosis (TB), along with other problems of public health importance in the country, were also affected. In 2001, the National Tuberculosis Institute (NTI) was the main government body providing diagnostic and treatment services with the collaboration of World Health Organization (WHO). According to the estimates, only 14% of the Afghan population had access to DOT services at that time. [8].

Post-2001, a National Tuberculosis Control Programme (NTP) was re-established. Services for tuberculosis patients were integrated into the basic health services being delivered at the primary care level [9,10]. Over the subsequent years, the country achieved the targets set by the Stop TB Department for National Tuberculosis Programmes [11]. With an improved rate of case finding and treatment success, the NTP achieved a gradual decrease in the incidence and TB-related mortality [12]. Moreover, the country’s own contribution to the NTP budgets started taking a positive direction.

In a country where healthcare delivery was crippled to the extent that it once reported the highest ever maternal mortality ratio [13], these achievements are remarkable. Moreover, the TB diagnosis and treatment services are fully integrated within primary care through the Basic Package of Health Services (BPHS) more completely than in many other countries where ‘vertical’ (disease-specific) approaches of programme delivery are used. What are the key processes that enabled the programme achieve its targets? More importantly, what are the underpinning factors that made this success possible? We addressed these important questions through a case study.

### Case description

National Tuberculosis Control Programme Afghanistan, with its successful implementation and impacts, was the “case” for this study. We adopted a processes and outcomes framework for this case study. We began by examining the change in key programme indicators with backwards tracing of the processes and underlying factors likely responsible for this change. WHO Afghanistan in collaboration with the NTP, and Stop-TB partnership at the Eastern Mediterranean Regional Office (EMRO) of the WHO developed the terms of reference for this study to examine the developments occurring during the period from 2001 to 2011. The information sources for this study included programme documents and articles published in peer-reviewed journals, followed by semi-structured interviews with key informants involved in the care of tuberculosis patients in Afghanistan.

The published and unpublished reports were provided by the NTP, while peer-reviewed articles were electronically searched using the key words Afghanistan, tuberculosis, conflict, program implementation, and lessons learned. The reviewed documents included annual reports of the NTP Afghanistan from the year 2006 to 2011, and reports of external review missions to the NTP Afghanistan which comprised expert members like GFATM, MSH, AKUH, USAID and WHO. Global TB reports published by the WHO from 2001 to 2012 and articles published in peer reviewed journals on health systems in conflict areas, especially services for tuberculosis patients in conflict and post-conflict situations, were also consulted. Progress toward targets and factors responsible for it, along with issues and challenges faced by health services in conflict and post-conflict situations were examined.

The key informants for interviews were selected on suggestion of the NTP as well as through snowball technique i.e. one interviewee suggesting additional stakeholders who could become potential key informants for this study. Interviews were conducted using semi-structured interview guides developed for this purpose. The interviewees included managers at the NTP and NTI, partners including the TB Patients Association and WHO, donor organizations including Italian Cooperation, Japan International Cooperation Agency (JICA), Canadian International Development Agency (CIDA), and United States Agency for International Development (USAID). Open ended questions were posed addressing how the respondents viewed the performance of the NTP in Afghanistan, factors responsible for successes and failures, and how the programme could address the current and future challenges.

A total of 15 interviews were carried out with respondents including NTP (3), WHO (4), USAID (2), Italian Cooperation (2), JICA (1), CIDA (1) and TB patient association (2). All invited respondents agreed to participate in the study. Interviews were held in the office of the respective respondents. Objectives of the study were explained to all the respondents before conducting the interview. Manual note-taking as well as digital voice recording of the discussions was done with the permission from the respondents. All but one of the respondents agreed to digital recording; only manual note-taking was done in that case. Transcripts of discussions were undertaken by combining the information from the digital recordings and manual notes. These final transcripts were treated as the data for analysis of the discussions. Data collection was completed from 12 June 2011 to 8 August 2011.

Initial analysis of the interviews began on the same day by reflecting on the transcripts. Final thematic analysis was carried out by reading and re-reading the compiled transcripts. Manual coding [14] of the transcripts was the first step towards data reduction. Patterns within the codes were summarized as sub-themes and displayed in the form of a matrix. Constant comparison technique [14,15] was used to evaluate how emerging sub-themes correlated or differed with each other. Finally, themes emerging from the similar sets of sub-themes were derived [14,15]. Respondent validation was ensured [16] by sharing the findings of the study with all stakeholders to have their comments. Confidentiality was practiced at every stage including anonymising the quotes from the interviews for this paper.

#### Programme achievements identified in the documents

The improvement in programme performance, reflected in the key programme indicators, is summarised in Table 1. The total number of notified TB cases increased from 9668 in 2001 to 28167 in 2011 and the percentage of successfully treated cases improved from 84 in 2001 to 90 in 2011 [17]. Moreover, drug resistance was prevented to keep multi-drug resistance (MDR) low (3.4% in 2011) and a low incidence (<1%) of TB-HIV co-infection was maintained [12]. The improvement reflected in the impact indicators with the incidence of TB (all forms) going down from 325/100,000 (2001) to 189/100,000 in 2011 and mortality from 92/100, 000 in 2004 (the earliest estimate available) to 39/100,000 in 2011 (Table 1). Non-availability of actual number of people that migrated during the period under study, and those that were treated under DOTS, increased our reliance on estimates which may not be the best representatives of the actual picture.

**Table 1:** Year wise (2001–2011) performance indicators of NTP Afghanistan

	**2001**	**2002**	**2003**	**2004**	**2005**	**2006**	**2007**	**2008**	**2009**	**2010**	**2011**
^1^Population (millions)	22	22	23	24	25	26	26	27	27	28	29
^2^Cases notified (yearly total)	9668	13551	13616	18385	21850	25474	28769	28301	26358	28238	28167
^3^Case detection (thousands)	28	35	34	43	50	55	61	53	48	47	46
^3^Incidence All cases (rate/100,000)	325	321	314	333	333	333	168	161	168	189	189
^3^Prevalence (SS+/100,000)	NA	NA	NA	302	671	661	288	231	238	352	351
^2^Treatment success rate (%)	84	87	86	89	90	84	87	88	87	90	91
^3^Mortality (rate/100,000)	NA	NA	NA	92	93	92	35	32	30	38	39
^3^MDR (% new cases with MDR)	NA	7.3	7.3	7.3	7.3	1.8	1.7	3.4	3.3	6.1	3.4
^3^HIV co-infected (% of all TB)	0.1%	0	0	0	0	0	0	0	0	<1	<1
^3^Funding total ($ millions)	NA	NA	2.8	1.3	1.8	4	2	9.5	10	6.2	5.3
^3^Domestic contribution ($ millions)	NA	NA	0	NA	0.3	0	0	0	0.5	0.6	0.7

^1^The World Bank Population Data 2014.

^2^National Tuberculosis Control Program Annual Report 2012.

^3^World Health Organization, Global TB Reports 2001–2011.

Despite poor security in the country, the NTP improved its performance with consistent hard work over the course of a decade. In 2001, TB services were seriously hampered due to the risks in certain areas of the country [8]. The government had adopted the directly-observed treatment, short-course (DOTS) strategy in 1997 as national policy, but only 14% of the population had access to services in the year 1999 [8]. The situation started changing when the Afghanistan’s Ministry of Public Health (MOPH) and its international partners including the WHO, USAID, JICA, CIDA, the Italian Cooperation, the European Commission and various non-governmental organizations (NGOs) made tuberculosis a national priority. The NTP was structured in 7 regions and 34 provinces in 2004-2005 [18]. This was followed by rapid expansion of services for diagnosis and treatment of tuberculosis, linked with the BPHS; the primary care system operating in the country.

Over the course of a decade, about 97% population of the country has access to DOT services being provided through more than 1000 health facilities [17]. There are 600 microscopy units, each of them providing sputum testing to an estimated 50,000 people living within their vicinity [17]. A recent study evaluated the surveillance system using the US Centres for Disease Control and Prevention guidelines. Usefulness and flexibility of the NTP system were found to be good; stability, representativeness and data quality were average; while simplicity, acceptability and timeliness were poor according to this evaluation. Positive predictive value and sensitivity were 11% and 70% respectively [19]. In addition to building the infrastructure, the NTP has especially focused on capacity building of its workforce at various levels. For example, a total of 1483 doctors and 1221 nurses were given initial or refresher trainings during 2010 [18]. Owing to their bridging role between community and health facility, community health workers (CHWs) are primary focus of capacity building, hence a total of 4131 CHWs were trained on DOTS administration during the same year [18].

The NTP has introduced uniform curricula and training systems incorporating, for example, Tuberculosis/Human Immunodeficiency virus (TB/HIV) and quality assurance (QA) guidelines. An efficient referral system has been created between NTP and National Aids Control Programme. A total of 6827 TB patients were referred for counselling and testing during 2011, out of them 2 were found to be HIV positive [17]. New initiatives like private-public mix (PPM) and TB/HIV are playing their respective roles of engaging semi-public and private practitioners into TB care, and integrating the TB and HIV diagnosis and treatment services. Highly populated and crowded settings with higher chances of TB e.g., jails are being specifically focused for inclusion into TB care services [17]. The TB patients association has been formed to involve and empower patients and communities, and implement the TB patients’ charter. The association is registered with Ministry of Justice and is a member of the Stop TB Partnership. It also has provincial associations in Herat, Kandahar, Mazar-e-Sharif and Jalal Abad.

Directly observed treatment (DOT) is provided by CHWs or nursing staff in health facilities. As most CHWs are women; this approach improves access to women, who bear a disproportionate share (70% women, 30% men) of the tuberculosis burden presumably due to high malnutrition and continuous living in crowded homes and refugee camps [20]. CHWs are instructed in the administration and follow-up of treatment. They are supported by community health supervisors at each facility and receive non-financial incentives, such as training and certificates. Poor patients who cannot meet their nutritional requirements receive food packages from the World Food Programme (WFP), although the logistical arrangements of this supply need improvement. A brief outline of various programme components enabled during 2001–2011 is provided in Table 2.

**Table 2:** Programme components enabled during the decade 2001-2011

**Programme component**	**Functions**
*Surveillance system*	Responsible for collection, analysis and interpretation of TB-related data, and timely dissemination to the stakeholders.
*Laboratory network*	600 laboratories; each covering the population of about 50,000. An External Quality Assurance (EQA) system is in place.
*Drug management*	Ensures uninterrupted supply of anti-TB drugs, MDT, and chemicals to all regions and provinces throughout the country. Also responsible for revision and updating of TB drug management and logistic system guidelines and their distribution to all provinces.
*Advocacy, Communication and Social Mobilization (ACSM)*	ACSM section is implementing a 5-year ACSM strategy to empower people and involve communities into TB care. Celebrating world TB days, meetings with policy makers, appearances on radio and television, and meetings with print and electronic media are the main advocacy activities. Public broadcasting through radio and television programmes, installation of billboards and activities at school and mosque level are the main communication and social mobilization activities.
*Health system trengthening*	The NTP has adopted a strategy in line with the policy of the Ministry of Public Health. The programme is integrated with BPHS and has SOPs for adoption by the BPHS staff.
*New Initiatives*	Development of policies and strategies, and monitoring of the implementation of new interventions such as Public-Private Mixed DOTS, TB/HIV co-infection, Multi Drug Resistant TB, and TB care for vulnerable groups are main responsibilities of this section.
*Stop TB partnership*	Includes 3 institutional mechanisms: the Secretariat, the Coordinating Board, and the Partners’ Forum. The Secretariat provides support to the partnership in terms of administration, operational implementation, and strategic decisions. The Coordinating Board comprises representatives from the NTP, the WHO, donors, academia, business sector, religious leaders, BPHS partners as well as communities and ad-hoc members. The Partners’ Forum is the assembly of the Stop TB Partnership and consists of inclusive, consultative meetings of representatives of all partner departments and organizations.

#### Factors underpinning the progress identified by the interviews

1. Resiliency of the programme

NTP Afghanistan has been dealing with challenges such as physical destruction of buildings, precarious security leading to frequent disruption of logistics, and psychological and emotional issues of both the staff, and the patients. The programme possesses enough resiliency and character to survive these difficulties. For example, the NTI building, the headquarters of the NTP, was built by JICA in 1978 but was destroyed during the war. JICA recommenced collaboration with the NTP during the post-Taleban period and renovated the building in 2002–2003. The NTP staff did not wait for long and started resurrecting their programme for efficient delivery of services as soon the basic level of facilities were available at the NTI building.

*“I still remember the time during 2002–2003 when there was no computer-based surveillance system. Even the facility to print or photocopy the data collection forms was not available, and the programme staff used to prepare forms by hand and then fill them with the required information.”-*NTP Official

Not only the programme but the patients too had to face diverse challenges while fighting their disease. Talking about the times before 2001, a cured TB patient shared:

*“Many times, roads would get blocked across the border of Afghanistan and Pakistan, causing shortage of TB drugs imported from Pakistan. This would mean many TB patients having to live without taking their medications, sometimes for weeks. I was one of them but I continued my battle till my disease was over.”-* Member from TB Patients Association

Because of the resiliency factor, the personal experiences of hardships resulted in higher motivation levels of some patients. Not only they completed their treatment, they went on to become volunteers who would help others in their area, as a member of the TB patients association.

2. Political commitment

The programme enjoys commitment from the highest political level. MOPH and its functionaries including the Minister for Health take interest in NTP which is crucial in ensuring commitment of the donor community. The commitment from higher levels trickles down to the lower cadre of health providers resulting in effective and sustained implementation of the programme.

*“I know a lot of professionals in the NTP are well trained and committed and have collaborated with the donors. Without such professionals, there is not much that the donors could do with their money.”-*International Partner

Talking about commitment of local people, one of the stakeholders said the NTP, without the donors, would still be able to continue at 70% of its current performance. Service delivery in Afghanistan, according to this informant, was not just a question of money but the vision and organizational commitment on the part of the programme. One anecdote shared by many partners was about the time when the entire programme staff worked for about nine months without salaries.

*“The fact that the programme, from top to bottom, worked for 9 months without salaries was amazing. This also happened with BPHS staff; they didn’t get salary for 4–5 months and continued their work. One can argue that they continued because they get much more than the ordinary government salary but perhaps it had more to do with their level of motivation than anything else.”-*International Partner

3. Partnerships

The Stop-TB partnership in Afghanistan is working for the elimination of TB from the country. Its vision is to secure a TB-free Afghanistan by ensuring that every TB patient has access to effective diagnosis, treatment, and cure. Partnerships have a critical role in achieving this vision.

*“NTP is getting good opportunities of funding because of the support available from its partners. We as a programme also feel responsible in such scenarios because the onus is on us to implement the ideas funded on proposals submitted by the programme.”-*NTP official

The respondents shared that sometimes the partnerships become a little complicated when objectives of the partners don’t match with those of the programme. The technical assistance from the WHO and the leadership skills of the NTP help the situation by matching the objectives of the programme and the partners. For example, CIDA had a strong component on gender sensitization and gender balance; rather than asking for funds for its existing components, the NTP developed a proposal on including a gender-sensitive dimension to the TB care in Afghanistan, that was approved.

4. Coordination mechanisms

According to the respondents, some effective coordination mechanisms through regular meetings and e-sharing have been established among donors helping to address the TB burden in Afghanistan. Because of these mechanisms, every partner knows what others are planning or implementing, which enhances the chances of complementing each other with little duplication of efforts.

*“I have visited several countries for TB control programmes. Some countries are not good in coordination. They don’t share information among the partners. Here it is different. Afghanistan has very good coordination mechanisms which help improve the programme effectiveness.”-*International Partner

Another level of coordination is achieved by involving patients, their families and communities through communication and social mobilization activities. Various influential individuals and groups like the religious scholars including *Mullah* and the *Pesh Imam* of the mosque are engaged through such activities. The TB Patients Association is another helpful initiative for coordinating community-based activities and forging partnerships at the community level.

5. Technical assistance and support

The discussions revealed that the support provided by partner countries coupled with the technical assistance (TA) role of the WHO played a significant role in effective implementation of the programme. While donor countries provided almost 100% of the annual budgetary support (Table 1), the WHO helped bring a convergence to these financial initiatives by steering the partnership among diverse players like CIDA, JICA, Italian Coop and USAID, and donors like the Global Fund for AIDS, Tuberculosis and Malaria (GFATM). Efficient coordination and information sharing mechanisms established for this proved crucial for avoiding the duplication of efforts and the waste of resources. In addition, the NTP did not have capacity in the initial days to fulfil grant requirements of donors like GFATM; the WHO helped the programme by providing technical assistance for proposal writing and fulfilling grant implementation requirements

#### Challenges indentified in the interviews

1. Coordination and service delivery issues in remote areas

The respondents highlighted the challenges of service delivery, given the security situation in remote areas. While acknowledging the integration of DOTS into primary care through BPHS, they also mentioned the implementation aspects like enhancing lab efficiency, provision of supplies, and improving counselling capacity of health providers in rural areas to improve the overall delivery of TB care services. They thought that BPHS had a massive infrastructure as compared to NTP, and further coordination and capacity building of the staff could help in improving DOTS delivery, especially to the populations living in far flung areas.

“*There is this NTP and then a much larger BPHS; coordination between the two needs improvement. The basic package which is the main delivery mechanism for populations living in rural areas does not have laboratories, technicians and diagnostic and treatment facilities at certain places. Patients in these areas may have to travel long distances which is not easy in Afghanistan. Some of them may overlook their symptoms or hide their sickness. Many of them might end up not availing diagnosis and treatment at all.” –*International partner

2. Lack of trained staff

Lack of adequately trained human resource in the distant locations emerged as an important challenge being faced by the programme. More frequently, the lack of trained female staff was mentioned as a challenge common to TB care, as well as to the primary healthcare delivery. The interviewees attributed this challenge to the prolonged war and conflict because of which the Afghan populations, especially females, could not attain adequate levels of education. Consequently, enough number of properly qualified female staff is not available to be trained and deployed by the healthcare system.

*“There is a lack of human resource, proper trainings and a lot of health facilities don’t have female staff. In the Afghan context, female staff is crucial because without females being around, the examination, treatment advising and health education to female patients becomes impossible.”-*NTP Official

3. Donor dependence and sustainability issues

While the participants described the current achievements, they also appeared cognizant of the issues of financial sustainability. Sustainability of the programme especially post-2014, when international support will be withdrawn or minimized, emerged as a recurrent theme. The programme managers seemed confident of the capability of the service delivery and mindful of the fact that ultimate financial sustainability would depend upon the Government of Afghanistan. They, however, thought that this would be a slow and evolutionary process and bilateral funding should phase out in a systematic manner rather than quitting abruptly. The partners agreed and added that the Government of Afghanistan’s list of prioritized programmes which included NTP was an important development and the Government needed to strengthen it through appropriate policy decisions and increased allocation of resources.

*“Now is the time that we should think about the time when international partners will have gone back home. The Government’s list of prioritized programmes is a positive development but it needs finalization and consolidation by enhancing resources for these programmes.”-*International partner

### Discussion and evaluation

Our review of the programme documents and interview with stakeholders of TB in Afghanistan shows that services for tuberculosis are being effectively carried out in major parts of the country. Despite the ongoing situation of war and conflict, the NTP has achieved a gradual improvement in case detection (seen by increasing case notification) and treatment outcomes (seen by increasing treatment success and decreasing mortality). At the implementation level, a near universal coverage of DOT services by integrating its diagnostic and treatment services to the BPHS through 600 microscopy centres and over 1000 treatment canters is a significant achievement [17]. An ongoing capacity development of staff working at various levels of healthcare in the country is also an important step towards effective implementation of the programme.

A number of key factors could be identified for the successful resurrection of the structures and functions of the programme. Political commitment from the MOPH, leadership from the NTP managers and dedication to work from the staff are foremost among these factors. Mauch et al. in their review of tuberculosis programmes in fragile states have commented that national programme leadership and stewardship are essential for quality and sustained TB control [10]. We agree and add that leadership and stewardship are essential in all contexts but crucial in conflict areas where many donors and stakeholders keenly want to contribute but cannot actively work in the field because of political and logistical reasons, and usually partner with local communities and programme for effective implementation.

The technical assistance role of the WHO appears significant in the context of Afghanistan. Even though the donors were pledging money in the post-war period starting from 2001, the core government functions were weak combined with a lack of public funding and human resources dedicated to health programmes. In post-conflict countries, weakened ministries of health do not have capacity to provide health services to their populations but they can coordinate with development partners and ensure that policies formulated by them are implemented [21,22]. In the case of Afghanistan, an agency was required that could assist the local programme and engage with donors and satisfy their funding requirements; the WHO effectively fulfilled this requirement.

The main challenges being faced by the NTP are logistic and security issues, coordination with other components of primary care within BPHS, lack of adequately trained human resource, and financial dependence on donors. Presently, these challenges are being met through strong political commitment to TB control, programme leadership, technical assistance by the WHO and a plan for gradual withdrawal of support from the donor countries. Our findings are consistent with other studies conducted on programme feasibility in fragile states [9,10,23,24].

To ensure equitable service delivery, the quality of services being provided at the grass roots level must be improved. Our desk review revealed that diagnostic sensitivity of the health workers for possible cases of pulmonary tuberculosis was low and needed improvement. In a study carried out in 24 health centres in 8 provinces, 22% patients with symptoms suggestive of tuberculosis did not receive a clinical diagnosis [25]. Moreover, prolonged delays between onset of symptoms and treatment provision have been recorded from certain parts of the country [26]. These factors highlight the need for improved practical training of health providers and health education of individuals and communities to improve programme impact.

TB patients are usually poor and malnourished, and more likely to have complications and mortality than the normally nourished patients [27,28]. The issue of poverty and consequent malnutrition and vulnerability to infection and its complications becomes paramount in a country like Afghanistan which is low-resourced and has undergone devastation for a long period of time [7]. These patients need both short- and long-term rehabilitation. The short-term problems are more acute and need urgent help including financial support to travel to health facilities, to buy food, and to ensure education of their children. NTP’s collaboration with WFP to provide food support to TB patients is a step in the right direction, however, this support should be provided in more organized and consistent manner. In addition, the NTP should engage organizations like United Nations Education, Scientific and Cultural Organization (UNESCO) to ensure education of TB patients’ children and consider initiatives like voucher schemes for monetary assistance of these patients to address basic needs such as travelling to and from health facilities.

Certain limitations to the study must also be mentioned. The actual numbers on certain programme indicators for the entire study period were not available and we had to rely on reported estimates for this study. For example, the high (>90%) treatment success rate [17] seems quite high for a country under conflict, and this rate could be explained better if actual numbers were available. Secondly, the key informants for this study were proposed by the NTP and their opinions could be biased towards the programme, although we tried to address it through additional snow-ball sampling. Lastly, due to time constraints and security issues, we could not interview the programme’s service delivery arm i.e., health care providers working at health facility and community level. Their views could have helped understand the programme’s implementation at the grass-root level in a better way.

## Conclusions

The NTP Afghanistan is a success story of effective programme implementation despite the poor security and war like situation that the country has faced on an ongoing basis. Having acknowledged the success, some issues and challenges also need to be mentioned. The foremost is effective delivery of services at the most peripheral level of healthcare services in the country. It is usual for governments in conflict settings and their partners to concentrate efforts on urban areas where there is some health infrastructure, and leave rural areas for coverage later [4,22]. The MOPH Afghanistan, on the other hand, adopted a bottom-up approach by focusing first on the primary care level, which is commendable [29,30]. However, the DOT services in rural areas need further consolidation to ensure a functioning, equitable health system that can have important health and state building benefits in a post-conflict country [22].

Public health programmes in conflict or post-conflict areas usually face the challenge of when and how to effectively transition from relief to consolidation and to development programming [31]. Disagreements occur on how best to strengthen weak governments, and prevent duplications that occur frequently. The WHO helped effectively address these challenges in the case of Afghanistan. The efficient coordination mechanisms and communication platforms like Stop-TB Partnership, frequent meetings of partners supplemented by an e-portal of information sharing, and community coordination through TB patients and religious leaders helped all partners have access to each other’s plans and priorities. It is now the responsibility of NTP to take the stewardship of coordination mechanisms and keep the partnership functioning at an optimum level.

The NTP Afghanistan is an example that public health programmes can be effectively implemented in fragile states. Given the high commitment and strong leadership, the programme can continue providing high quality services to Afghan populations, manage the current challenges, and move on to the phase of consolidation and sustainability. To ensure its long-term effectiveness, the withdrawal of international support should be a slow and evolutionary process, and bilateral funding should phase out in a systematic manner rather than quitting abruptly. At the same time, the MOPH needs to enhance its financial contribution to the NTP budgets to ensure the structural and functional sustainability of the programme.

## Abbreviations

AKUH: Agha Khan University Hospital; BPHS: Basic Package of Health Services; CIDA: Canadian International Development Agency; DOT: Directly observed treatment; DOTS: Directly-observed treatment, short-course; EMRO: Eastern Mediterranean Regional Office; GFATM: Global Fund for AIDS, Tuberculosis and Malaria; JICA: Japan International Cooperation Agency; MOPH: Ministry of Public Health; MSH: Management Sciences for Health; NGO: Non-Governmental Organizations; NTI: National Tuberculosis Institute; NTP: National Tuberculosis Control Programme; PPM: private-public mix; QA: quality assurance; TB/HIV: Tuberculosis/Human Immunodeficiency virus; TA: technical assistance; TB: tuberculosis; UNESCO: United Nations Education, Scientific and Cultural Organization; USAID: United States Agency for International Development; WHO: World Health Organization.

## Competing interests

The authors declare that they have no competing interests.

## Authors’ contributions

Kh S, contributed to conception, design and acquisition, analysis and interpretation of data, and was involved in drafting the manuscript. DE, contributed to analysis and interpretation of data, and was involved in revising the manuscript. KS, contributed to acquisition, analysis and interpretation of the data. ZH, contributed to conception, design, acquisition of data and writing/revision of the manuscript. WK contributed to design, analysis and writing stage of this manuscript. All authors approved the final manuscript for publication.
